# Using Text Messaging Surveys in General Practice Research to Engage With People From Low-Income Groups: Multi-Methods Study

**DOI:** 10.2196/55354

**Published:** 2024-09-05

**Authors:** Elizabeth Sturgiss, Jenny Advocat, Christopher Barton, Emma N Walker, Suzanne Nielsen, Annemarie Wright, Tina Lam, Nilakshi Gunatillaka, Symrin Oad, Christopher Wood

**Affiliations:** 1 School of Primary and Allied Health Care Monash University Frankston Australia; 2 Department of General Practice School of Public Health and Preventive Medicine Monash University Melbourne Australia; 3 Monash Addiction Research Centre Eastern Health Clinical School Monash University Melbourne Australia; 4 Department of Health Victorian State Government Melbourne Australia; 5 Australia and Melbourne School of Global and Population Health University of Melbourne Melbourne Australia; 6 EPIC Research Unit School of Primary and Allied Health Care Monash University Frankston Australia; 7 School of Primary and Allied Health Care Monash University Melbourne Australia; 8 Capital Health Network Canberra Australia

**Keywords:** SMS, data collection, research methods, disadvantaged population, priority populations, message, messages, messaging, disadvantaged, underserved, survey, surveys, digital divide, marginalized, access, accessibility, barrier, barriers, smartphone, smartphones, digital health, underrepresented, data collection, mobile phone, short message service

## Abstract

**Background:**

SMS text messages through mobile phones are a common means of interpersonal communication. SMS text message surveys are gaining traction in health care and research due to their feasibility and patient acceptability. However, challenges arise in implementing SMS text message surveys, especially when targeting marginalized populations, because of barriers to accessing phones and data as well as communication difficulties. In primary care, traditional surveys (paper-based and online) often face low response rates that are particularly pronounced among disadvantaged groups due to financial limitations, language barriers, and time constraints.

**Objective:**

This study aimed to investigate the potential of SMS text message–based patient recruitment and surveys within general practices situated in lower socioeconomic areas. This study was nested within the Reducing Alcohol-Harm in General Practice project that aimed to reduce alcohol-related harm through screening in Australian general practice.

**Methods:**

This study follows a 2-step SMS text message data collection process. An initial SMS text message with an online survey link was sent to patients, followed by subsequent surveys every 3 months for consenting participants. Interviews were conducted with the local primary health network organization staff, the participating practice staff, and the clinicians. The qualitative data were analyzed using constructs from the Consolidated Framework for Implementation Research.

**Results:**

Out of 6 general practices, 4 were able to send SMS text messages to their patients. The initial SMS text message was sent to 8333 patients and 702 responses (8.2%) were received, most of which were not from a low-income group. This low initial response was in contrast to the improved response rate to the ongoing 3-month SMS text message surveys (55/107, 51.4% at 3 months; 29/67, 43.3% at 6 months; and 44/102, 43.1% at 9 months). We interviewed 4 general practitioners, 4 nurses, and 4 administrative staff from 5 of the different practices. Qualitative data uncovered barriers to engaging marginalized groups including limited smartphone access, limited financial capacity (telephone, internet, and Wi-Fi credit), language barriers, literacy issues, mental health conditions, and physical limitations such as manual dexterity and vision issues. Practice managers and clinicians suggested strategies to overcome these barriers, including using paper-based surveys in trusted spaces, offering assistance during survey completion, and offering honoraria to support participation.

**Conclusions:**

While SMS text message surveys for primary care research may be useful for the broader population, additional efforts are required to ensure the representation and involvement of marginalized groups. More intensive methods such as in-person data collection may be more appropriate to capture the voice of low-income groups in primary care research.

**International Registered Report Identifier (IRRID):**

RR2-10.3399/BJGPO.2021.0037

## Introduction

Mobile phone ownership in countries with advanced economies is almost universal [1,2], and most phone users regularly send text messages through an SMS. [3] An SMS text message is one of the most frequently used channels of interpersonal mobile communication that enables real-time exchange of alphanumeric messages, commonly in packages of up to 160 characters [4], and may be delivered manually or through an automated system.

SMS text messages have been used for clinical and research purposes with promising results in terms of patient feasibility and acceptability. While the standard route for collecting patient data in health research has traditionally been paper-based surveys [5], SMS text message methods may offer equivalent, if not higher, response rates than paper-based methods [6,7], and patients express a preference for SMS text message–based surveys over paper-based alternatives [8], including in low-income settings [9]. When information from multiple time points is requested (as in the case of daily or weekly surveys), SMS text message–based methods may offer a greater chance of obtaining more reliable, complete data, as recall bias can be minimized [6]. In addition, SMS text messaging may assist in collecting data on stigmatized topics, with some evidence suggesting that participants are more likely to disclose issues, such as mental health and substance use information, when asked by non–paper-based methods (eg, SMS text message and internet surveys), rather than face-to-face interviews [10,11].

Surveys of patients in primary care often have low response rates, and this rate is even lower among disadvantaged groups [12]. This reduced participation in research occurs for a variety of reasons, including financial barriers to participation, language barriers, and lack of discretionary time [12]. A recent case study conducted in Australia found that SMS text message surveys of Arabic-literate participants recruited through a community group were successful in data collection while recognizing difficulties with translating materials into readily understandable Arabic resources [13].

Digital inclusion is an important consideration for improving research participation [14]. Digital technologies have been highlighted as a potential option for low-cost, scalable solutions for survey participation that could allow adequate representation of participants from disadvantaged groups. There is variation in access to digital technology through device type, and disadvantaged communities may find the internet most accessible through mobile phones [15]. A recent Australian study of surveys conducted with a culturally and linguistically diverse group using SMS text messages recommended further research on using SMS text messaging surveys with people from low-income groups [13]. There continue to be disparities in smartphone ownership, limited access to data or Wi-Fi, and language barriers that can impede participation in digital health initiatives [16]. While SMS text messaging surveys appear to be an attractive option for research data collection, more research is needed to determine if this would allow equitable participation to achieve digital inclusion [16].

We aimed to explore the use of SMS text messaging for recruitment and data collection in general practice research with a focus on patients who are socioeconomically disadvantaged.

## Methods

### Ethics Approval

The study was approved by the Monash University Human Research Ethics Committee (22865). Participants provided informed consent to participate. Data were deidentified.

### Recruitment

This substudy was nested within an implementation trial of the REACH (Reducing Alcohol-Harm in General Practice) project [17,18]. The overall objective of the study was to explore the feasibility and acceptability of a tool kit to support the use of alcohol brief interventions in general practice. This paper focuses exclusively on the use of SMS text messages to collect data from patients through general practice.

This study was set in Melbourne, Australia. Within Australia, 83.6% of the population is estimated to visit a general practitioner (GP) at least once a year [19], and smartphone ownership is estimated to be 91% [20]. The general practices were located within the state of Victoria, which is Australia’s second-largest jurisdiction by population. To be eligible for this study, the general practice had to be situated in a lower socioeconomic area [18]. There were 6 general practices located in lower income communities that participated in the REACH trial and were recruited for the implementation trial through the local primary health network (PHN) [18]. PHNs are independent, primarily federally funded organizations across Australia that support primary health care providers, including general practices and commission services based on local unmet needs. They are similar in function to other primary care commissioning bodies in the United Kingdom and Canada [21].

### SMS Text Message Data Collection

Each practice had its own process for sending the initial SMS text message, details of which are outlined in the Results section. Practices were paid an honorarium of US $658 (US $1=Aus $1.52) to cover some of the costs associated with sending the SMS text messages, including administrative time. The research program manager and PHN staff were also available to discuss any issues the practice had with sending SMS text messages and followed up practices that were having technological difficulties. This approach meant that the research team did not have access to patient names or phone numbers at any time.

There were 2 main steps to the SMS text message surveys. For the first step, a staff member in each practice was asked to send an SMS text message to all current patients aged 18 years or older through an SMS text message blast (where an SMS text message is sent out to a large group of numbers at one time). This initial email contained a link to an online Qualtrics survey (Textbox 1). Patients needed a smartphone to accept the SMS text message as well as mobile data or access to a Wi-Fi connection to complete the survey online. All surveys were in English.

**Initial SMS text messages sent to patients by their general practice.**

“Hello, you are receiving this message as you recently visited <<Practice Name>>. We and Monash University are inviting you to complete a survey on alcohol and your health through <<Survey link>>. The survey is confidential, voluntary, and will help us improve the service we deliver to you. Thanks!”

To include patients who attended during the implementation of the REACH project, patients were eligible to complete the online survey if they had visited the general practice within the past 3 months. At the end of the online survey, patients could elect to give their details (name and mobile phone number) for further SMS text message surveys to be sent 3, 6, and 9 months to capture data about their alcohol use and whether they had recently consulted with their GP (Multimedia Appendix 1). We did not have formal checks to assess the authenticity of responses or remove duplicates within Qualtrics. However, the recruitment process (direct SMS text message from the patients’ GP clinic) and lack of an incentive to provide multiple responses make it unlikely that we had bot-generated or multiple responses.

The second step involved 2-way SMS text message surveys sent to patients who submitted their details in the online Qualtrics survey that was then downloaded and stored in a password-protected secure drive. In a 2-way SMS text message survey, it is only possible to ask closed questions that can be answered with a number (Textbox 2). The 2-way SMS survey had 5 questions in total and each question had 5 possible responses that were indicated by the patient selecting a number between 1 and 5. It should be noted that patients did not have any contact with the research team between the 3-month, 2-way SMS text message surveys.

**Two-way SMS text message surveys were sent to patients from the research team who agreed to receive the surveys in Step 1.**

Hello! This is the REACH team.

Thank you for agreeing to take part in our survey when you last visited your doctor. Text STOP to opt-out of this survey (Note–the respondent answers the SMS with the number that corresponds to their answer)

- Q1 How often do you have a drink containing alcohol?
  - Never (1)
  - Monthly or less (2)
  - Two to four times a month (3)
  - Two to three times a week (4)
  - Four or more times a week (5)
- Skip To: Q4 If the answer is (1), Never
- Q2 How many standard drinks do you have on a typical day when you are drinking?
  - One or two (1)
  - Three or four (2)
  - Five or six (3)
  - Seven to nine (4)
  - Ten or more (5)
- Q3 How often do you have six or more standard drinks on one occasion?
  - Never (1)
  - Less than monthly (2)
  - Monthly (3)
  - Weekly (4)
  - Daily or almost daily (5)
  - Q4 Have you spoken with your GP in the last 3 months?
  - No (1)
  - Yes, and we spoke about alcohol (2)
  - Yes, but we didn't talk about alcohol (3)
  - I can't remember (4)
- Q5 Which of the following describes you?
  - I'm drinking within safe limits (1)
  - I plan to cut down in the next 3 months (2)
  - I want to cut down but haven't decided when (3)
  - I should cut down but really don't want to (4)
  - I don't want to cut down (5)

### Interviews and Qualitative Analysis

Toward the end of the REACH trial (May to August 2021), JA, an experienced qualitative research fellow, interviewed PHN staff, clinicians, and practice staff at each of the general practices about their experience of participating in the REACH project, including the SMS text message substudy reported here. JA is a qualitative researcher and an allied health clinician, with more than a decade of experience in primary care research. All staff and clinicians of the practices who were involved with REACH were invited to participate. Data collection concluded when the authors had interviewed participants from each practice and had sufficient data to explain the process of implementation.

The semistructured interview guide included questions about the benefits and barriers of using SMS text messages to communicate with patients. The interviews were conducted remotely due to COVID-19 restrictions (phone or videoconference), and lasted from 18 to 60 minutes, with most approximately 30 minutes. They were audio-recorded and professionally transcribed. The excerpts of the interviews pertaining to SMS text messages were used in the analysis of this specific study. The elements related to SMS text messages were coded inductively using NVivo software (version 14, QSR International) and then organized according to constructs from the Consolidated Framework for Implementation Research (CFIR) [22] codebook to understand the findings in relationship to implementation factors, both in the inner and outer contexts of the practices.

JA verified the transcripts and coded the data, and a subset of investigators formed an analysis team who met regularly (JA, ES, SN, TL, NG, and CB) to discuss findings based on the CFIR factors.

## Results

### Overview

The practices involved in the REACH project were all located in lower socioeconomic areas and most practices estimated that more than 50% of their patients lived in low-income households [18]. Only 4 of the 6 participating practices were able to send the initial survey link by SMS text message to their patient cohort. One practice reported that IT problems meant they were not able to send SMS text messages to their patients at all, and they did not use SMS text messages to communicate with their patient group. A second practice felt that the SMS text message was inappropriate as their patient group was highly culturally and linguistically diverse. The practice preferred a poster in their waiting room with a QR code for the online survey and then they could assist patients to complete the survey or explain the survey, as needed. However, no patients from this practice completed the survey through the QR code.

Each of the 4 general practices that were able to send SMS text messages to their patients had very different internal processes and thus ended up sending the SMS text message to vastly different numbers of patients. One practice could send the SMS text message to each current patient’s mobile number in their system, and a total of 5286 SMS text messages were sent, with 506 commencing the survey (9.6%). Another practice could only send the SMS text message to patients attending on the actual day due to an IT system issue, so they sent it to 500 patients over 3 days with 8 responses (1.6%). The third practice had software that did not allow any past patients to be contacted (usually not seen within 2 years). This practice sent an SMS text message to 2500 patients with 172 initial responses (6.9%). The final practice required patients to give their specific consent to receive the SMS text message, so they asked patients as they presented to the practice and only sent it to those who agreed, with a total of 50 sent across 2 days with 16 responses (32%).

**Figure 1:**
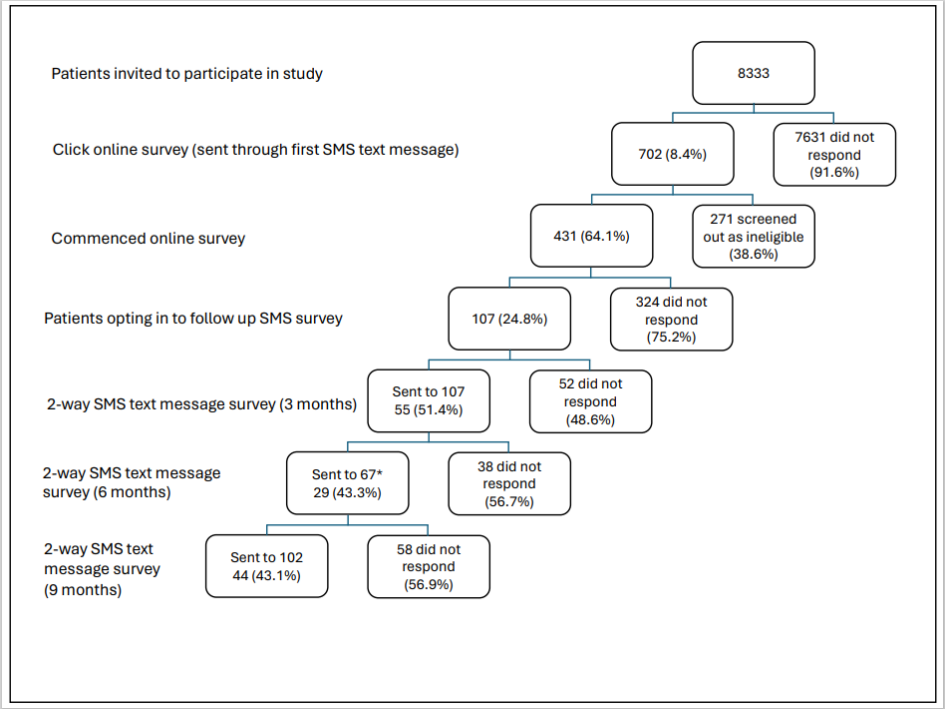
Number of SMS text messages sent and response rate at each step of the SMS survey. *The 6-month 2-way SMS text message was sent to 67 participants; 40 participants only received the 2-way SMS survey at 3 and 9 months.

Figure 1 shows the overall response rate at each step of the SMS text message study. In total, 8333 SMS text messages were sent by the practices, and 702 (8.4%) of SMS text messages received a response. To record a response, the patient had to click on the online link for the survey. This would require the mobile phone number for the patient on record to be current, and the mobile phone to have data or Wi-Fi to enable a response if they did click on the external link.

A total of 431 patients completed at least some of the online surveys with a majority of the survey respondents being women, aged 45 years or older, and were not from a low-income group (Table 1).

Of the 431 survey respondents, 107 (24.8%) agreed to receive an SMS text message survey every 3 months. For the second step of the study using 2-way SMS text messages, the response rate improved to approximately 50% (216/431) for each survey (Figure 1).

**Table 1:** Respondents in the online survey. The survey link was sent through an initial SMS text message from the general practice (n=431).

Demographics			Participants, n (%)
**Age (years;** **n=340)**			
	18-24	15 (4.4)	
	25-34	27 (7.9)	
	35-44	50 (14.7)	
	45-54	86 (25.3)	
	55-64	70 (20.6)	
	65-74	74 (21.8)	
	75-84	16 (4.7)	
	85 or older	2 (0.6)	
**Gender** **(n=340)**			
	Woman	227 (66.8)	
	Man	105 (30.9)	
	Nonbinary or third gender	3 (0.9)	
	Prefer to self-describe as transgender male or nonbinary trans	4 (1.2)	
	Prefer not to answer	1 (0.3)	
**Low-income status (n=335; multiple choices possible)**			
	Unemployed and looking for work	17 (5.1)	
	Receive a government pension	50 (14.9)	
	Health care cardholder	16 (4.8)	
	Live in a low-income household	8 (2.4)	
	None of these apply to me	236 (70.4)	
	Prefer not to say	8 (2.4)	
**Do you have a chronic disease? (n=344)**			
	Yes	141 (41.0)	
	No	195 (56.7)	
	Prefer not to say	8 (2.3)	
**Did you talk to your doctor or nurse about your alcohol intake during your last visit? (n=377)**			
	Yes	58 (15.4)	
	No	303 (80.4)	
	Do not recall	15 (4.0)	
	Prefer not to say	1 (0.3)	
**How often do you have a drink containing alcohol? (n=344)**			
	Never	60 (17.4)	
	Monthly or less	77 (22.4)	
	2-4 times a month	138 (40.1)	
	4 times a week or more	69 (20.1)	

### Qualitative Findings

We interviewed 12 representatives from the 5 practices. At least 2 staff members were interviewed from each practice. Our sample included 4 GPs, 4 practice nurses (PNs), and 4 administrative staff members, including PMs. Table 2 summarizes the demographic and professional characteristics of participants.

A total of 7 PHN staff were interviewed, detailed in Table 3.

**Table 2:** Interviewees from each of the 5 general practices.

Practice		Professional background
**1**		
	Participant 1	General practitioner
	Participant 2	Practice manager
	Participant 3	Practice nurse
**2**		
	Participant 1	General practitioner
	Participant 2	Practice nurse
	Participant 3	CEO
**3**		
	Participant 1	General practitioner
	Participant 2	Administrative staff member
**4**		
	Participant 1	General practitioner
	Participant 2	Practice nurse
**5**		
	Participant 1	Practice manager
	Participant 2	Nurse/care coordinator

**Table 3:** Interviewees from the primary health networks.

Number	Staff role
1	Practice relationship manager
2	Practice relationship manager
3	Continuous quality improvement program officer
4	Project coordinator
5	Continuous quality improvement program officer
6	Continuous quality improvement program officer
7	Manager

In the interviews, the PMs and clinicians gave details about their experience of using SMS text messages in their own practice. PHN staff described any issues that came up with practices they supported that were relevant to the SMS substudy. Qualitative results are organized with reference to CFIR, to assist in understanding factors that affected the implementation, both internal and external, of the practices (Textbox 3).

**Findings from qualitative data mapped to the Consolidated Framework for Implementation Research constructs.**

**Outer setting**

- Needs and resources of the patient population, including language, tech ability, Wi-Fi access
- Competing demands from external policy, including those related to the COVID-19 vaccination roll-out

**Inner setting**

- Size of the general practice
- Networks and communication between team members
- Implementation climate
- Readiness for implementation of the SMS text message surveys

**Characteristics of individuals**

- Individual attitudes toward the SMS text message surveys

**Innovation characteristics**

- Complexity of the SMS text message process
- Relative advantage compared with usual ways of communicating with patients
- Cost-effective
- Modern forms of communication

### Outer Setting

#### Needs and Resources of Those Served by the Organization

Patient demographics came up often during interviews with clinicians and practice staff, including language spoken, age, cultural background, and mental health status. Concerns about patient literacy were reported, especially by staff at clinics serving more diverse communities:

I think from the patient’s perspective, there were some struggling, because we have 80% non-English speaking people here, so I think they struggled with the text messages.

P1GP

For patients whose primary language was other than English, it was common that they were neither able to read words written in their primary language, nor English.

…they’re not translated at all, even the SMS messages we were able to send out to the patients, we didn’t use them because it would just be them ringing us up saying, “We received a text message and we don’t know what it is.” So, it was just too much work, so we didn’t go ahead with that either.

P1PM

Patient characteristics also came up in relation to the patient population served at one of the practices, which included people with dual diagnoses of complex mental illness and alcohol and other drug addictions. It was thought by the PM that patients in this practice would not respond to SMS text messages and that they would not have the technical skills to adapt to new systems.

not everyone is tech-savvy. I have to say, some of my patients still have the flip-flop phones, not smartphones. So if we send - it definitely can receive a text message, but it's not adaptable to QR codes, to links, those things…So for us, a good old paper that they can take and read and not print out I think is still the best…

P5PM

If patients did have a suitable phone, they may not have a phone plan with data or access to Wi-Fi. Practices did not report providing free access to Wi-Fi for patients within their practice. Low phone credit could also make returning an SMS text message survey problematic.

The age of patients also came up as a relevant factor for 2 of the practices (P4 and P5):

For some, the younger ones prefer technological things, like SMS, QR codes. But the older ones prefer something to read that’s actually printed. Yeah. It’s a good thing that either way, you’ll be available for that.

P5PM

#### External Policy and Incentives

External policy and incentives is a broad CFIR construct that is related here to the inclusion of policy and regulations from the governmental level or external mandates. Given the timing of the study, one practice was rolling out the COVID-19 vaccination at the direction of the government at the same time they were trying to troubleshoot IT issues and did not have the resources to participate (from PHN2).

### Inner Setting

#### Structural Characteristics

The most prominent structural characteristic mentioned by participants was the size of one of the clinics. Its small size, having only 1 GP, meant that they had not set up the SMS text message component of the clinical software.

We don’t have a big amount to do, if you know what I mean. So, it’s just something that we never really set up, because we didn’t really need to use it. Like, when we go through our recalls, I just give it to reception and they just call each patient individually.

P4PN

#### Networks and Communication

Another practice found that networks and communication within the practice were not strong enough to maintain a “whole of team” approach. They were not, therefore, able to keep the SMS text message process in their institutional memory.

one of the other girls was involved, like organising the SMS’ to be sent out, but she’s actually left the clinic to take on another job.

P2PN

#### Implementation Climate

Here we examined the capacity for change of the practices. In total, 2 practices had work processes that lacked compatibility with the innovation. As 1 (PN) put it, their “IT guy” provided an “ongoing battle” to get it set up. (P4PN) The other practice noted that their clinical software was not compatible with what the study asked of them (P5PM).

#### Readiness for Implementation

Readiness for implementation refers to “tangible and immediate indicators of organizational commitment to its decision to implement an innovation.” The level of resources that an organization allocates to implementation is an indication of readiness. The CFIR subcode “available resources” came up in the data through a discussion of cost. In total, 1 PHN participant noted that the use of SMS text messages costs more than email and might have been a barrier to practices participating in this substudy.

“sometimes they’re not too keen on doing huge campaigns on SMS, because it actually costs them money to send the information. Whereas, via email would have been better, but it’s just difficult to pull that sort of information from the clinical software systems.”

PHN1

#### Characteristics of Individuals

An individual stage of change refers to an individual attitudes toward innovation. In total, 1 PHN participant noted that GPs are sometimes hesitant about change.

But a lot of them can be a bit hesitant to sending patients out anything, How will that look? Am I targeting my patients? They’re a bit anxious.

PHN6

#### Innovation Characteristics

In total, 1 PHN worker noted that one of the practices found the complexity of the intervention a bit more than they were used to and it made participation difficult:

I did have a practice that was struggling a little bit with – because there was the SMS surveys to send out. So I had a practice that was – they weren’t very used to using that sort of system. They just did phone recalls and reminders to patients, not SMS.

A total of 2 “Innovation Characteristics” were found to drive participation, including relative advantage and another way of understanding cost. In total, 1 practice in particular understood the SMS text message substudy offered them a couple of advantages over their usual practice. First, the SMS text message approach was thought to be “proactive and opportunistic,” enabling them to use a flexible approach to engage their “really passive” patients (P5PM). Second, the study was thought to provide improved access, giving the practice another alternative to offer patients:

I could give you this, I could send this to you via email. I can send it to you by text. So that adds to what we can do, what we can provide.

P5PM

For 1 PHN worker, the SMS study was a more modern and cost-effective approach to communicating with their patients:

I think they realised that it’s another way of communicating with the patients that they really should have been using for a long time. So that’s been quite a good benefit for that practice certainly, just getting them on board with a more modern way of working with their patients.

PHN1

#### Suggestions for Future Implementation

Practice managers and clinicians had several suggestions for how researchers could more successfully engage with patients who are from disadvantaged groups. These included preferentially using paper-based surveys that were administered at a place that patients already knew and trusted; having researchers available to assist patients in completing surveys if literacy, manual dexterity, or vision was a problem; and vouchers for patients who complete surveys (suggested US $6.6 [US $1=Aus $1.52]).

## Discussion

### Principal Findings

We found that sending an SMS text message through general practice did not lead to a high response rate from patients, but a 2-way SMS text message survey to patients who had answered the first SMS text message had a higher response rate. Our approach did not capture participants from more marginalized groups due to constraints related to technology and human factors.

We used 2 processes to explore the use of SMS text messages for data collection in general practice research (1) an SMS text message from the patient’s own general practice that contained an external link to an online survey and (2) a 2-way SMS survey that was sent from the research team to patients who agreed to be contacted. We found most general practices experienced technical constraints in sending SMS text messages, such as limited software infrastructure. A researcher needed to be proactive in communicating with the clinic’s professional and administrative staff to adapt each clinic’s unique practice processes to the SMS text message survey procedure. We also used a poster in the waiting room of one practice at the practice’s request; however, no patients used this QR code and it did not prove to be a practical strategy in this culturally and linguistically diverse patient group.

Only a small number of participants clicked the external link to the online survey within the initial SMS text message, but it was a comparable percentage to primary care surveys in general [12]. The ongoing 2-way SMS text message surveys were more successful as the patients had agreed to be contacted again and were able to answer questions within the SMS text message environment. Response rates remained over 50% for the 3-month SMS text messages, with no additional contact from the research team or the general practice.

CFIR provided a structure to tease out characteristics that influenced the successful uptake of the SMS text message study from the qualitative data [22]. The most relevant outer setting factors that impacted participation included the needs and resources of the patient populations, notably English language proficiency, age, and 1 practice, which worked primarily with addiction. The data further indicates that size, teamwork, and capacity for change all influenced the uptake of the SMS text message study.

While SMS text message surveys are an attractive option for primary care research, there are constraints to consider. These include a restriction in the type of questions that can be asked due to the limits of SMS text message length and closed question format. Furthermore, SMS text messages are unlikely to capture socioeconomically disadvantaged populations who have barriers, including phone infrastructure and hesitancy around unknown contact numbers. With a recent increase in SMS text message use in scams, this hesitance may increase in the future [23]. Researchers should also be aware that practices are likely to have their own policies or software limitations in sending SMS text messages to patients, and this is likely to influence recruitment and response rates, as seen in this study.

### Comparison With Previous Work

The lower response rate in our study is still in line with the international literature where response rates for SMS text message data collection vary considerably, from 12.5% to 100%, with the lowest uptake (12.5%) in a drug and alcohol clinic [24], while response rates beyond 90% were seen a decade ago in general practice, especially when youth were involved [25-27]. A patient’s willingness is likely dependent on a number of factors, including familiarity with texting, relationship with the individual recruiting them, health-related motivation or interest in research, and the presence of incentives [28]. Most longitudinal SMS text message surveys also see response rates typically decline, by an estimated 2%-13%, over the duration of the study, which is comparable to our study’s decline of 8% from baseline to the 9-month follow-up [24,29-31]. In studies where participants did not respond to an initial SMS text message, high responsiveness was seen after reminder messages were sent [5,32]. Other studies have called participants who did not respond to prompts, which was an effective method of retaining participants in the study, although this does increase the research staff’s time and requires consent for contact by phone call [8].

We kept our 2-way SMS text message survey as short as possible to improve response and completion rates [10]. Among patients who ceased surveys before completion or stated they would not be willing to complete a future text survey, a commonly cited reason was that the initial survey had too many questions [29,33]. More than half (52%) of patients in a Singaporean study stated they preferred surveys with 1-10 questions, and only 12% stated they would be willing to complete an SMS survey with more than 30 questions [29]. In 1 study, participants were asked what they would consider an acceptable number of SMS text messages to receive in 1 week from researchers, with a mean response of 4 (SD 3.7) [24], while most participants in another study felt 2 SMS text message questions per day was sufficient [9].

This study included about one-third of participants from a low-income group. The digital divide recognizes easier digital health access for the more advantaged patients [34], and patients have reported the cost of texting as a reason not to participate in an SMS text message trial [24]. The type of phone a patient uses may also influence completion, with patients who use smartphones more likely to complete SMS text message surveys than those using older, more basic phones [33]. With these recognized barriers, this study also highlighted the additional research effort that should be afforded to capture data from the most marginalized patients.

### Limitations

A key limitation of this study is the absence of qualitative data from patients about their experience of SMS text message surveys. Although clinicians and PMs could draw on their broader experience, they may have made some assumptions about patient preferences that we could not verify with patients themselves. Our low response rate, while a finding in itself, limited what we were able to learn about SMS text message surveys in lower income populations. We used the general practice as a trusted source to deliver the initial SMS text message, with the assumption that this would increase the response rate, but this did not appear to facilitate SMS text message responses. We are also unable to report if SMS text messages were actually received, which may artificially lower the response rate. For example, this could occur if the patient had changed their phone number since they were last at the practice. Our quantitative data could not make direct comparisons with those from other groups, and further work is required to determine if other strategies may make SMS text message survey work feasible in lower income primary care populations.

### Conclusions

While SMS text message survey methods offer a low-intensity option for research data collection, a general SMS text message survey is unlikely to capture participants from more marginalized groups. When recruiting patients through general practice, researchers need to consider the different practice protocols that may be in place for contacting patients by SMS text message as this greatly influences the potential of the method. To promote research participation from the most socially disadvantaged groups, paper-based, or researcher-facilitated surveys undertaken at a trusted location may yield more responses.

## Appendix

Multimedia Appendix 1 Reducing Alcohol-Harm in General Practice project online patient survey.

## Abbreviations

CFIR: Consolidated Framework for Implementation Research
GP: general practitioner
PHN: primary health network
PM: practice manager
PN: practice nurse
REACH: Reducing Alcohol-Harm in General Practice
