# Supplementary material for: Using Text Messaging Surveys in General Practice Research to Engage With People From Low-Income Groups: Multi-Methods Study
Source: JMIR Mhealth Uhealth. 2024 Sep 5;12:e55354. doi: 10.2196/55354 (PMC11413543; doi:10.2196/55354)
Supplement: Multimedia Appendix 1 [file mhealth_v12i1e55354_app1.docx]

## Supplementary file 1

REACH Project: Online Patient survey

Start of Block: Default Question Block

Q1 **Would you like to take part in a research project?**   You received this survey because you may have recently visited [Medical Practice}.    The practice is taking part in a research study. Doctors (GPs) and practice nurses at the practice are being encouraged to ask their patients more regularly about alcohol as part of the study.    We're interested in your views on doctors asking about your drinking.    If you are 18 years or older, please take a few minutes to fill in this survey.   Please read our [Explanatory Statement](https://www.monash.edu/__data/assets/pdf_file/0011/2179154/Explanatory-Statement-Patients-1.pdf) and contact the research team if you have any questions before starting the survey.

Q1 Have you visited this general practice clinic in the last 3 months?

- Yes (1)
- No (2)

Skip To: End of Survey If Have you visited this general practice clinic in the last 3 months? = No

Q2 Do you usually come to this general practice clinic when you need advice about your health?

- Yes (1)
- No (2)
- Unsure (3)
- Prefer not to say (4)

Q3 Who did you see at your last visit to the practice?

- My usual doctor (1)
- Another doctor (2)
- My usual nurse (3)
- Another nurse (4)
- Other, please specify (5) __________________________________________________
- Unsure (6)
- Prefer not to say (7)

Q4 How long have your known the doctor or nurse you saw that day?

- Meeting them for the first time today (1)
- This is the second appointment I have ever had with them (2)
- Less than one year (more than one appointment before) (3)
- About 1-5 years (4)
- More than 5 years (5)
- Prefer not to say (6)

Q5 Is the doctor or nurse you saw that day your preferred doctor or nurse?

- Yes (1)
- No (2)
- Prefer not to say (3)

Q6 What are the reasons for your consultation? (Select all that apply)

- To find out what is wrong / get a diagnosis (1)
- For reassurance (2)
- To get the results of test / investigations (3)
- For treatment (prescriptions, procedures) (4)
- For a routine check (5)
- For review (6)
- To ask for a referral (7)
- Other, please specify (8) __________________________________________________
- Prefer not to say (9)

Q7 Did you talk to your doctor or nurse about your alcohol intake **during your last visit**?

- Yes (1)
- No (2)
- Don't recall (3)
- Prefer not to say (4)

Display This Question:

If Did you talk to your doctor or nurse about your alcohol intake during your last visit? = Yes

Q8 Who started talking about alcohol?

- I did (1)
- My doctor or nurse did (2)
- Don't recall (3)
- Prefer not to say (4)

Display This Question:

If Did you talk to your doctor or nurse about your alcohol intake during your last visit? = Yes

Q9 How comfortable were you when talking to your doctor or nurse about alcohol?

- Very comfortable (1)
- Somewhat comfortable (2)
- Neutral (3)
- Somewhat uncomfortable (4)
- Very uncomfortable (5)
- Don't recall (6)
- Prefer not to say (7)

Display This Question:

If Did you talk to your doctor or nurse about your alcohol intake during your last visit? = Yes

Q10 Did you doctor or nurse offer you any of the following?
(Select all that apply)

- A discussion about how to reduce alcohol intake (1)
- Pamphlets and other written materials about alcohol (2)
- Self-help workbooks about alcohol (3)
- Details of websites, apps and other electronic resources about alcohol (4)
- Referral to specialist services e.g. AOD counsellor, addiction specialist (5)
- Other (about alcohol), please specify (6) __________________________________________________
- None of the above (7)
- Prefer not to say (8)

Display This Question:

If Did you talk to your doctor or nurse about your alcohol intake during your last visit? = No

And Did you talk to your doctor or nurse about your alcohol intake during your last visit? = Don't recall

And Did you talk to your doctor or nurse about your alcohol intake during your last visit? = Prefer not to say

Q11 Has your doctor or nurse **ever** talked to you about your alcohol intake?

- Yes (1)
- No (2)
- Don't recall (3)
- Prefer not to say (4)

Q12 Do you have any suggestions for how discussions about alcohol with your doctor or nurse could be improved?

________________________________________________________________

________________________________________________________________

________________________________________________________________

________________________________________________________________

________________________________________________________________

End of Block: Default Question Block

Start of Block: Block 1

Q13 **The next three questions describe how people might interact with their doctor or nurse. Thinking about your last visit to the practice:**

Q14 Did the doctor or nurse listen carefully to you?

- Yes (1)
- No (2)
- Prefer not to say (3)

Q15 Did they show respect for what you had to say?

- Yes (1)
- No (2)
- Prefer not to say (3)

Q16 Did they spend enough time with you?

- Yes (1)
- No (2)
- Prefer not to say (3)

End of Block: Block 1

Start of Block: Block 2

Q17 **These next questions about are about your use of alcohol. Your answers will remain confidential so please be honest.**

Q18

Q19 How often do you have a drink containing alcohol?

- Never (1)
- Monthly or less (2)
- 2-4 times a month (3)
- 2-3 times a week (4)
- 4 times a week or more (5)

Q20 How many standard drinks do you have on a typical day when you are drinking?

- 1 or 2 (1)
- 3 or 4 (2)
- 5 or 6 (3)
- 7 to 9 (4)
- 10 or more (5)

Q21 How often do you have six or more standard drinks on one occasion?

- Never (1)
- Less than monthly (2)
- Monthly (3)
- Weekly (4)
- Daily or almost daily (5)

Q22 **The last few questions are about your health and personal circumstances.**

Q23 Do you have a chronic disease?

A chronic disease is an illness that lasts for more than 6 months and can lead to your health getting gradually worse if the illness is not well managed. If you have chronic disease you may need to have a range of treatments and follow special lifestyle advice.   
Some common chronic diseases are: diabetes, arthritis, chronic depression, chronic pain, heart disease, chronic lung problems.

- Yes (1)
- No (2)
- Prefer not to say (3)

Display This Question:

If Do you have a chronic disease? A chronic disease is an illness that lasts for more than 6 months... = Yes

Q24 Do you have any of the conditions below? (Select all that apply).

- High blood pressure (1)
- Depression or anxiety (2)
- Musculoskeletal condition (3)
- Arthritis (4)
- Osteoporosis (5)
- Chronic lung condition (e.g. asthma, COPD, chronic bronchitis) (6)
- Heart disease (angina, ischaemic heart disease, peripheral vascular disease) (7)
- Heart failure (8)
- Stroke or TIA (9)
- Reflux, or gastric ulcer, or peptic ulcer (10)
- Bowel disease (IBS, ulcerative colitis, Crohn’s disease, diverticulosis, diverticulitis) (11)
- Chronic hepatitis (12)
- Diabetes (type 1 or 2) (13)
- Thyroid disease (14)
- Cancer in the last 5 years (including melanoma, but excluding other skin tumours) (15)
- Kidney disease or kidney failure (16)
- Chronic urinary tract infections (17)
- Dementia or Alzheimer’s disease (18)
- High cholesterol (19)
- Obesity (20)
- Other, please specify (21) __________________________________________________
- Prefer not to say (22)

Q25 How old are you?

- 18-24 years (1)
- 25-34 years (2)
- 35-44 years (3)
- 45-54 years (4)
- 55-64 years (5)
- 65-74 years (6)
- 75-84 years (7)
- 85 years and over (8)
- Prefer not to say (9)

Q26 What is your gender?

- Woman (1)
- Man (2)
- Non-binary/third gender (3)
- Prefer to self-describe (4) __________________________________________________
- Prefer not to say (5)

Q27 Do any of these apply to you?
(Select all that apply)

- Unemployed and looking for work (1)
- Receive government pension (2)
- Healthcare card holder (3)
- Live in a low income household (4)
- None of these apply to me (5)
- Prefer not to say (6)

End of Block: Block 2

Start of Block: Block 3

Q44 **Would like to take part in more research?**    We invite you to take part in an interview with a member of the research team and/or respond to an SMS survey at three monthly intervals after your visit to your doctor or nurse. You will be asked to tell us more about your experiences of talking to your doctor or nurse about alcohol.    Please fill in the consent form below and provide you contact details if you wish to participate.    For more information, please read our [Explanatory Statement](https://www.monash.edu/__data/assets/pdf_file/0011/2179154/Explanatory-Statement-Patients-1.pdf). If you have any questions, please get in touch with the research team.

Q45
 I am interested in taking part in the Monash University research project specified above. I have read and understood the Explanatory Statement and I hereby consent to participate in the following activities (please select all that apply).

- Taking part in an interview (with audio recording) (2)
- Taking part in an SMS survey at three monthly intervals (3)

Q46 Please provide your contact details below

|  | Contact details (1) |
| --- | --- |
| Name (1) |  |
| Email address (2) |  |
| Mobile phone number (3) |  |

End of Block: Block 3
